# It’s not what you expect: feedback negativity is independent of reward expectation and affective responsivity in a non-probabilistic task

**DOI:** 10.1007/s40708-016-0050-6

**Published:** 2016-04-18

**Authors:** Jonathan M. Highsmith, Karl L. Wuensch, Tuan Tran, Alexandra J. Stephenson, D. Erik Everhart

**Affiliations:** 1 Department of Psychology, East Carolina University, 238 Rawl Building, East 5th St., Greenville, NC 27858 USA; 2 Mulidisciplinary Studies Program in Neuroscience, East Carolina University, East 5th St., Greenville, NC USA

**Keywords:** Affect regulation, Decision making, Cortical asymmetry, Feedback negativity

## Abstract

ERP studies commonly utilize gambling-based reinforcement tasks to elicit feedback negativity (FN) responses. This study used a pattern learning task in order to limit gambling-related fallacious reasoning and possible affective responses to gambling, while investigating relationships between the FN components between high and low reward expectation conditions. Eighteen undergraduates completed measures of reinforcement sensitivity, trait and state affect, and psychophysiological recording. The pattern learning task elicited a FN component for both high and low win expectancy conditions, which was found to be independent of reward expectation and showed little relationship with task and personality variables. We also observed a P3 component, which showed sensitivity to outcome expectancy variation and relationships to measures of anxiety, appetitive motivation, and cortical asymmetry, although these varied by electrode location and expectancy condition. Findings suggest that the FN reflected a binary reward-related signal, with little relationship to reward expectation found in previous studies, in the absence of positive affective responses.

## Introduction

### Overview

Although being studied for over a century [1], reward-based learning remains an area of investigation to help understand human decision making. While some might argue that understanding the cognitive aspects of this process is unnecessary [2], reward learning inherently requires an executive control cognitive process: online monitoring of behavioral performance. A consistent view of the systems thought to regulate decision making and online performance monitoring includes the anterior cingulate cortex (ACC) [3–5], basal ganglia [6], and prefrontal cortex (PFC) areas as a network that performs planning and complex decision making [7, 8]. Electrophysiological investigations focused on this performance monitoring commonly involve eliciting an event-related potential (ERP) characterized by a negative deflection in the waveform over frontal scalp areas, termed the feedback negativity (FN), which is suggested as a component of a general and flexible system involved in overall error-related processing [9].

Source localization studies suggest the FN is generated in the ACC [10–15], which is supported by functional neuroimaging [16–21]. The ACC is a nexus of inputs and outputs with links to numerous cortical and subcortical structures, including being a portion of the limbic system and having interconnectivity with many prefrontal areas [22, 23]. Reinforcement learning (RL) theory proposes that error-related ERP components reflect ACC responses through its connection to the mesencephalic dopamine system, via deviations from predicted expectations [24]. Many prefrontal cortex areas are also associated with encoding of expectancy and reward outcomes, suggesting that cortical areas are involved in addition to the dopamine reward system [8, 25–27]. Early studies suggested that the frontal error ERPs corresponded only to valence of outcomes [24, 28, 29], while later studies demonstrated that both valence and magnitude of outcomes influenced FN signals [15, 30–32]. Alternately, various studies have shown that valence, magnitude, and probability are all involved in prediction errors, or the difference between expected and actual rewards, and that FN deflections are related to the overall probability of reward outcomes [10, 16, 30].

The ACC is also directly involved in the conscious experience of emotions [3], decision making related to affective behaviors, affective motor programs [33] and as the primary cortical area for both emotion reception and emotional motor output [34]. The ACC is further implicated as the affective processing center in that abnormal ACC activity has been associated with depression, and that depressed participants show abnormal responses to errors within the rostral ACC [35], with attenuated FN responses in severely depressed individuals [36, 37]. This implicates the ACC in both general performance monitoring and affective processing, and suggests that the FN may be influenced by both bottom–up and top–down cognitive and affective processes.

### Feedback negativity and individual differences

While these basic cognitive and affective processes may influence the expression of the FN, research on individual differences in reward sensitivity has also been theorized to play a role in general approach and withdraw behaviors and responses to affective stimuli, which are linked to trait levels of resting activation in the frontal cortices [38–40]. A number of studies have identified that frontal activity is related to approach and withdraw behaviors and increased responsiveness to valenced affective stimuli [39, 41–43]. Subcortical inputs from periaqueductal gray and septo-hippocampal systems have also been posited to influence reinforcement sensitivity via individual differences in a behavioral activation system (BAS) and behavioral inhibition system (BIS), leading to greater sensitivity to certain types of stimuli and general response tendencies, shown through surface level trait impulsivity and anxiety [34]. These effects may be evident in the BIS/BAS constructs and their relationship to personality variables such as impulsivity, reward responsiveness, and sensation seeking and individual differences in risk taking behavior during gambling tasks [44] and feedback negativity [45]. The frontal asymmetry model shows some overlap with the BIS and BAS model, but there is a significant disparity in that measures of BAS and increased trait left frontal activity show consistent agreement, while any links between BIS and increased right frontal activity are less clear [38, 46–49].

Many researchers have already completed significant work on isolating factors influencing FN responses to stimuli. Investigation of the ACC responses to feedback commonly involves gambling tasks with expectancy induced through variations in outcome probability [10, 27, 30, 50–53] with the FN showing larger amplitude responses for unpredicted outcomes [51]. Within many of these studies, researchers used varying reward probabilities as an index of reward expectancies, although there is considerable evidence that people do not have rational beliefs about randomness, probabilities, nor gambling [54–56]. There are even indications that the ACC is directly involved during uses of fallacious reasoning [57]. Thus, we cannot simply assume rational expectation of reward based on neither previous stimulus reinforcement nor direct variation of apparent probability, particularly within gambling type tasks.

Bellebaum and Daum [10] found that the FN showed increased amplitude with unexpected outcomes and the magnitude between the expectation and actual rewards, but that the effect did not appear until after the subjects successfully learned the probability rule. Bellebaum et al. [30] showed that the FN response coded for valence, magnitude in the non-rewarded trials, and higher expectancy for those learning the task and concluded that the FN coded the total degree of departure from expectancy along all three elements (valence, magnitude, and probability). De Pascalis et al. [46] found a relationship between the FN and BIS; however, the task involved the continuous winning of money and induced significant increases in positive affect in all participants, being further enhanced in those subjects reporting high reward responsiveness. Van den Berg et al. [58] used a gambling task to elicit a FN, but found no association between the FN and reward responsiveness, while Lange et al. [45] found that FN elicited by unexpected outcomes was associated with higher BAS. More recently, Bress and Hajcak [59] found that gambling-related FN amplitudes were larger for individuals with higher self-reported reward sensitivity and behavioral reward sensitivity on a signal detection task.

### Hypotheses

The current study was proposed to contribute to this literature by allowing for investigation of the impact of expectancy on amplitude of FN responses in relative isolation from probabilistic-based task limitations, while attempting to limit possible affective task responses. This required subjecting participants to a boring pattern response learning task. The induction of varying expectancy, between high and low expectancy conditions, was based largely on presentation of the task as a simple executive function (memory of small sequences). It was hypothesized that participants would not demonstrate affective response or report mood changes associated with the task, and that the FN would not show a significant negative deflection based on participant expectancy of losses in the absence of task-induced affect responses.

## Methods

### Participants

Eighteen healthy, adult undergraduates (10 women) with a mean age of 20.4 years (SD = 2.8) participated and were provided class credit. All participants were right-handed and reported normal or corrected vision with no history of neurological or psychological disorders. Subjects were rescheduled if they reported receiving less than 5 h of sleep the night before, or reported use of drugs or alcohol within 48 h of the study, or report use of any other substance that might interfere with alertness or electrophysiological measurement. No participants reported symptoms suggestive of problem gambling behavior on the South Oaks Gambling Screen (SOGS) [60]. In order to limit any monetary remuneration influence on participant selection, only following reporting for the study and disclosing no disqualifying information were participants informed that they would be entered in a raffle for a $50 cash prize, with their performance during the task determining the number of entries. All participants provided written informed consent prior to the study, which was approved by the University and Medical Center Institutional Review Board.

### Measures

Participants completed the Stroop Neuropsychological Screening Test (SNST) [61] and confirmed non-impaired ranges of incongruence processing and attention with *T* scores from 42 to 99 (*M* = 72.44, SD = 22). Participants also completed Carver and White’s [62] BIS and BAS scales prior to psychophysiological recording. After preparation for recording, participants completed two self-report questionnaires measuring both trait and baseline affect and anxiety consisting of the State-Trait Anxiety Inventory—Form Y (STAI) [63] and the Positive and Negative Affective Schedule (PANAS) [64]. The PANAS measure was repeated following completion of the procedure. Additionally, subjects were instructed to complete Self-Assessment Manikin (SAM) scales measuring affect and arousal, following completion of each six series within the experimental procedure as an additional within-task verification of affect and arousal response.

### Procedure

Participants were briefed regarding the experimental procedures and were explicitly told that the correct choices were not random, but came in four different response patterns, and to utilize patterns to maximize winnings. They were also informed that these patterns would shift throughout the procedure. Participants were seated in a dimly lit acoustically shielded room approximately 1 m from a LCD stimulus display. They were instructed to limit movement during baseline and task measurements, and were verbally directed to close or open eyes for each minute of baseline recording. Following baseline recording procedures, participants were directed to comfortably arrange a four key stimulus keypad in both hands, with their thumbs positioned on the outside two of the four keys. The stimulus display presented directions regarding the responses, specifically to use the thumb of their left hand to select the left card and the thumb of their right hand to select the right card. Participants were given the opportunity to conduct two practice series to familiarize themselves with the game. These series consisted of two trials following the pattern of alternating win and loss feedback.

**Fig. 1:**
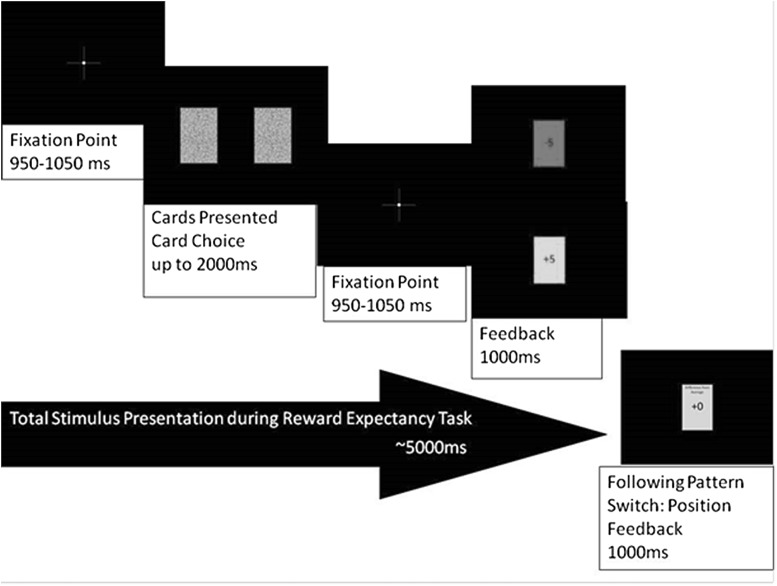
Series of stimuli across task

Each task trial totaled approximately 5 s in length, as shown in Fig. 1, and began with a 1-s presentation of a fixation point followed by the presentation of two textured rectangles (roughly mimicking a playing card) equally spaced horizontally on the LCD screen, which remained until participants selected one of the two rectangles. Following selection, the fixation cross reappeared for one second after which a feedback rectangle appeared, centered on the LCD screen, filled green for win and red for loss with the number of points won or lost centered in the middle of the rectangle (+5 or −5). Feedback was presented for one second, followed by the reappearance of the fixation cross for the next trial. However, following a shift in correct response pattern, the feedback presentation was followed by additional presentation of current total winnings and their difference from the average of all participants on that series presented in another rectangle for 1 s each prior to the reappearance of the fixation cross. As the task required little cognitive load, the scores showed little variability with feedback of +5 to −5 points of the average presented in all cases, intended to limit any affective response due to consistent wins experienced.

Each presented pattern consisted of a 2 trial learning phase with mixed win–loss feedback, with a subsequent period of 6–15 consistent wins based on randomly determined length of pattern repetition and participant performance. This consistent period was designed to increase expectancy of win feedback during the period, the middle trial of which was taken to represent the high expectancy win trial. Participants were given a loss feedback to signal a pattern shift and represented the high expectancy loss trial. The subsequent trials in which participants were guessing the new pattern consisted of both win and loss feedback, which represented the low expectancy win and low expectancy loss trials. Variation between number of trials per pattern and selection of the middle pattern trial was conducted to prevent the possible confound of the participants expecting a pattern shift in later win trials and thus demonstrating decreasing win expectancy as pattern shifts became more likely due to a high number of consistent wins. Thirty pattern shifts were presented with an average number of 13 trials per pattern and a total task time of approximately 45.5 min, with five breaks following each 6 pattern shifts for SAM scale completion. Total participant time burden was approximately 90 min.

### Electrophysiological recording

Cortical electrical activity was recorded using Ag/AgCl—sintered electrodes mounted in an elastic Quik-Cap (Compumedics Neuroscan; Herndon, VA) at 32 scalp sites using the international 10/20 placement system (Fp1, Fp2, F7, F8, F3, F4, FT7, FT8, FT9, FT10, T3, T4, FC3, FC4, C3, C4, CP3, CP4, TP7, TP8, T5, T6, P3, P4, O1, O2, Fz, FCz, Cz, CPz, Pz, Oz) with a ground electrode placed on the frontal aspect anterior to electrode Fz, and referenced to linked ears (A1–A2/2). The impedance of all electrodes was maintained at less than 5 kΩ prior to acquisition. Electro-ocular (EOG) activity and eye blinks were acquired simultaneously and continuously using pairs of Ag/AgCl electrodes placed lateral to the lateral canthus of each eye for EOG, and placed superior and inferior to the center of the left eye for eye blinks. All measurements were acquired using a 40-channel NuAmps DC amplifier and NEUROSCAN ACQUIRE 4.4 (Compumedics Neuroscan), using a 512 Hz sampling rate within the 0.1–50 Hz frequency band. EOG and eye-blink ocular artifact reduction transformation occurred offline using a multiple lag time domain regression analysis [65, 66].

EEG baseline was collected using eight 1-min recordings using alternating eyes-open and eyes-closed periods. Following eye-blink and EOG corrections, each 1-min period was transformed from the time to frequency domains using overlapping 2.048 s epochs extracted through a Hamming Window with contiguous epochs overlapping 75 %, and then power spectra were calculated using a fast Fourier transformation and summed across all recordings as outlined in suggested recording guidelines [67]. Total alpha power between 8 and 13 Hz was examined and assumed to be the inverse of cortical activity [40, 68]. Power spectral data were confirmed to be positively skewed, prompting use of a natural log transformation to normalize the spectral data prior to averaging across all baseline measures. Asymmetry scores were computed as ln(right)-ln(left) for each electrode pair, so that higher scores are indicative of increased right alpha power and thus inferred to represent greater left cortical activity [40].

**Fig. 2:**
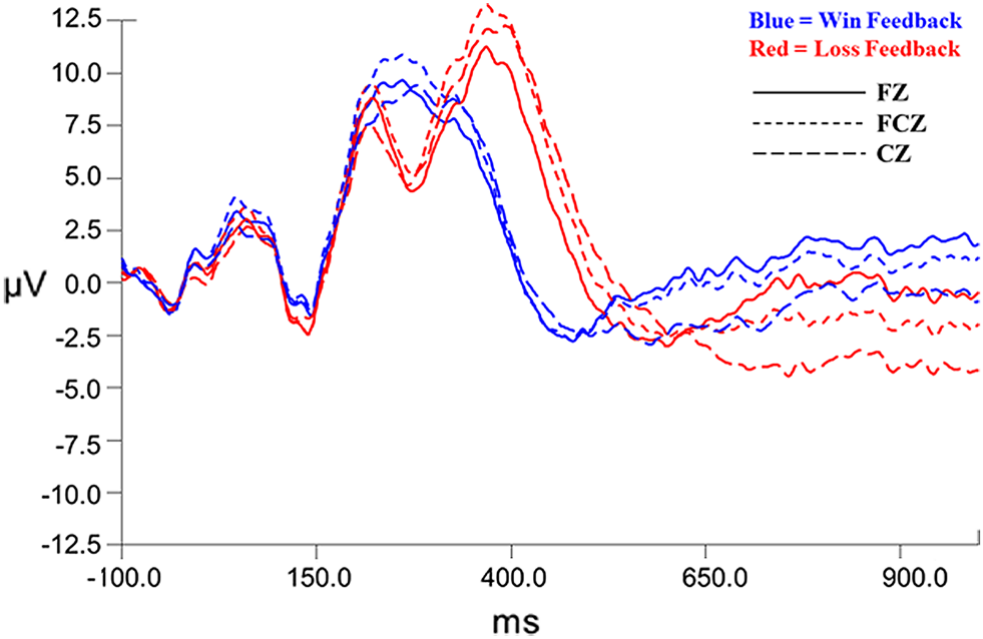
Grand average waveforms for anterior midline electrodes

ERPs were extracted from continuous measurements using 1100 ms epoch windows, including a 100 ms pre-feedback baseline and the 1000 ms following time locked feedback presentation in the eye-movement corrected data [69]. All files were digitally filtered using a 24 db low pass filter and baseline corrected relative to the 100 ms pre-feedback baseline. Time course windows for the FN were determined relative to computed grand averages, as shown in Fig. 2. The FN amplitudes were extracted as the most negative peak in the time window between 225 and 325 ms following feedback presentation. Grand average waveforms revealed an obvious large positivity occurring after the FN-type response in the high win expectancy condition, around 400 ms, and a large positivity occurring about 400 ms post feedback presentation in difference waves for both high and low expectancy conditions. Although not planned a priori, this prompted further investigation of the component, which was referred to as the P3 in subsequent analyses. As there were no planned analyses for this ERP component, it was decided to simply duplicate the analyses selected a priori for the FN component.

Separate average waveforms were obtained for four identified experimental conditions (High Expectancy Loss, High Expectancy Win, Low Expectancy Loss, and Low Expectancy Win). The High Expectancy Win condition was taken from the fourth epochal feedback window during consistent win presentation signaling correct pattern establishment and, presumably, a higher expectation of subsequent win feedback. The High Expectancy Loss condition was taken from the epochal feedback window following the first incorrect trial following consistent pattern establishment, signaling a pattern shift to the participant. The Low Expectancy Win condition was taken from the epochal window following the first correct trial following a pattern shift, while the Low Expectancy Loss condition was taken from the window following the first incorrect trial following the loss signaling a pattern shift.

## Results

### Demographics

Of the screening and collected demographic information, only age and education level were significantly positively related; this relationship was marginal (*r* = .476, *p* = .046). Participant age was also found to be significantly positively related to PANAS positive affect scores assessed following completion of the task (*r* = .699, *p* = .001). Stroop *T* scores were found to be significantly positively related to STAI Trait Anxiety scores (*r* = .569, *p* = .014).

### Performance data

Participant average response times ranged from 416 to 629 ms (*M* = 498.7, SD = 61.78). Participant scores ranged from 1635 to 1900 points (*M* = 1818, SD = 70.52), but was negatively skewed (*g*_1_ = −1.717) due to the presence of two outliers with a large number of response time errors, defined as a subject response within 100 ms of stimulus presentation, showing a positive skew (*g*_1_ = 1.961). Due to the departure from normality in the score and response time errors accounting for almost all of the score variance (*r* = −.971, *p* < .001), overall score was not utilized in later analyses. Due to the positive skew in the response time error data, a log transformation was utilized to induce normality. These log-transformed response time errors were significantly related to an overall tendency to respond quickly in pattern response decision making (*r* = −.648, *p* = .004), and subject Stroop *T* scores (*r* = −.564, *p* = .015), suggesting that these response time errors may be a behavioral indicator of impulsive responding. In addition, average response times were significantly related to BAS-Drive (*r* = .524, *p* = .025) and BAS Total (*r* = .484, *p* = .042) such that individuals endorsing a high level of drive to achieve rewards spent more time making response decisions, as well as fewer errors (*r* = −.515, *p* = .029). Of note, however, is that a high number of response time errors were also related to increased baseline negative affectivity (*r* = .590, *p* = .01), suggesting that perhaps individuals presenting with higher state negative affect tended toward lower overall investment in the task and/or more impulsivity in task-related decision making, which could also explain the correlation between response time errors and Stroop *T* scores reported earlier. For the anterior asymmetry measures, only the F4–F3 electrode pair was significantly related to response time errors (*r* = −.550, *p* = .018) so that greater relative left frontal activity was related to fewer response time errors.

### Affective response to task

As the response pattern learning task with win feedback consisting primarily of points was selected in an effort to limit possible affective responses to the task, a number of analyses were conducted to identify any within task affect variation. Within subjects repeated measures ANOVA on within task visual-analog scales indicated a significant reduction in positive affect across the five blocks, *F*(4, 14) = 3.673, *p* = .009, MSE = 2.15, *η* ^*2*^ = .21. A trend analysis indicated that the data were well fit by a linear model accounting for a significant portion of the variance in affect (*η* ^*2*^ = .19, *p* = .017). A second within subjects repeated measures ANOVA on within task visual-analog scale of arousal showed that arousal was not significantly changed over the course of the task, *F*(4, 14) = 1.070, *p* = .378, MSE = 2.06, *η* ^*2*^ = .04. This suggests that our task did not eliminate affective responses and somewhat negatively affected overall subjective affect with a boring task, while overall arousal was not significantly affected.

From the perspective of affect as two separate constructs, paired samples *t* tests on PANAS pre and post measures revealed that positive affect was significantly diminished over the course of the task, *t*(17) = 4.459, *p* < .001, *d* = 1.18, while negative affectivity increased, but not to the level of statistical significance, *t*(17) = 1.503, *p* = .151, *d* = 0.39. Although designed to be affectively neutral, the pattern of significant affect response to the task appears to be a decrease in positive affectivity, but not a significant increase in negative affectivity or general arousal. As reported earlier, participant age was significantly positively related to PANAS positive affect scores at posttest, but not at baseline. This suggests that the older, more senior student participants showed less of an emotional response to the task and showed greater preservation of positive affect, possibly due to greater acceptance of experiment participation in general, and not simply due to the design of the task itself. Regardless, these findings suggest that affective responses may be having an effect on feedback processing.

### ERP analysis

**Fig. 3:**
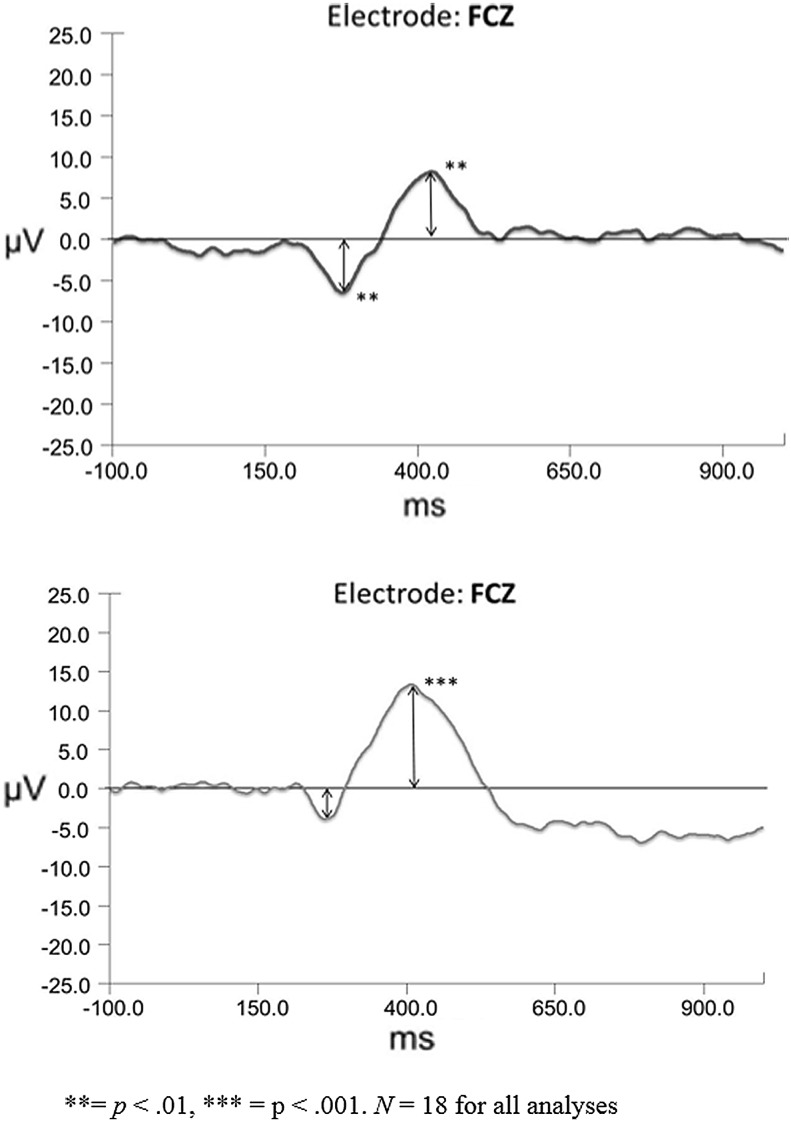
Difference waveforms for loss–win for low win expectancy (*top*) and high win expectancy

In order to determine if an FN response was induced between positive and negative feedback, win and loss grand average waveforms were created for each expectancy condition. Paired *t* tests were conducted on the FN amplitudes extracted for each of the frontocentral electrode points. Difference waves were constructed as a subtraction of the loss condition grand average minus the win condition grand average with a representative waveform for the FCZ electrode shown in Fig. 3.

A negative deflection in processing of feedback was found in the low win expectancy condition with amplitudes being significantly lower for loss versus win feedback for the FZ electrode, *t*(17) = 2.519, *p* = .022, SEM = 0.925, *d* = 0.60, and for the FCZ electrode, *t*(17) = 3.041, *p* = .007, SEM = 0.821, *d* = 0.72, but not for the CZ electrode, *t*(17) = 1.994, *p* = .062, SEM = 0.887, *d* = 0.48. Comparisons of component latency revealed no significant differences between conditions for FZ, *t* (17) = 0.173, *p* = .87, SEM = 9.64, *d* = 0.05, FCZ, *t*(17) = 0.476, *p* = .640, SEM = 9.58, *d* = 0.12, or CZ, *t*(17) = 1.963, *p* = .066, SEM = 13.13, *d* = 0.47. Thus, the significant amplitude differences between the loss and win feedback were identified in the more frontal electrodes, with no differences in latency, suggesting an FN-type response. Win and loss grand average waveforms were also constructed for the high win expectancy conditions. There was a negative deflection occurring in loss feedback processing within the established FN time window: however, amplitude differences were not statistically significant for FZ, *t* (17) = 0.921, *p* = .37, SEM = 1.74, *d* = 0.22, for FCZ, *t*(17) = 1.185, *p* = .252, SEM = 1.58, *d* = 0.28, or for CZ, *t*(17) = 1.347, *p* = .196, SEM = 1.50, *d* = 0.37 suggesting that either a significant FN-type deflection was not yielded, or no true FN was manifested in the high win expectancy condition.

We tested our main hypothesis that the FN would not show a significant negative deflection based on participant expectancy of losses in the absence of task-induced affect responses, through use of a one-sample *t* test on computed FN amplitude difference scores (low win expectancy difference—high win expectancy difference), for the FZ, FCZ, and CZ electrodes. As shown in Table 1, for all three electrode sites, FN amplitude differences were not significantly different from zero.

**Table 1:** One-sample *t* tests of FN component difference scores

Measure	Diff (μV)	*t* value	Sig (2-tailed)	SEM
FN FZ	.726	.496	.626	1.464
FN FCZ	.637	.439	.666	1.451
FN CZ	−.247	.178	.860	1.385

*N* = 18 for all analyses

### Personality analysis

While the study was powered for the above ERP analyses, exploratory analyses were conducted on personality measures to identify any additional indications of possible associations with theorized motivation systems. As these analyses suffer from reduced power, non-significant associations were not interpreted. With the BAS subscales, the derived total score was significantly correlated with only BAS-Drive (*r* = .820, *p* < .001) and BAS Fun Seeking (*r* = .650, *p* = .003), with BAS reward responsiveness being non-significantly negatively correlated with BAS Fun Seeking (*r* = −.311), resulting in the BAS total score being dropped from later analyses as it likely represented a non-unitary measure with limited interpretive utility. Of the anterior alpha asymmetry measures, the F4–F3 electrode pair was significantly correlated with scores on the BAS-RR scale (*r* = .471, *p* = .048), suggesting that overall greater left cortical activity is associated with reward seeking behavior and increased reward sensitivity. STAI trait anxiety scores were significantly positively correlated with the Behavioral Inhibition Scale (*r* = .648, *p* = .004). PANAS negative affectivity scores were also significantly related to BIS scores (*r* = .571, *p* = .013). Anterior alpha asymmetry was significantly related to baseline state anxiety on lateral frontal electrodes (*r* = −.648, *p* = .004) and negative affect (*r* = −.497, *p* = .036), so that greater relative left frontal activity was associated with decreases in both state anxiety and negative affect at baseline, which is intuitive in light of the positive relationship found of left frontal cortical activity and measures of appetitive motivation. Not surprisingly, STAI trait anxiety was significantly negatively correlated with BAS Fun Seeking scores (*r* = −.517, *p* = .028), while scores on behavioral inhibition were also significantly related to BAS Fun Seeking (*r* = −.633, *p* = .005). STAI state anxiety was significantly negatively related to baseline positive affectivity scores (*r* = −.481, *p* = .043), supporting the view of PANAS scores as a state variable.

### Reward sensitivity/affect and ERPs

Higher P3 amplitude differences for the FZ electrode were significantly related to higher trait anxiety (*r* = .474, *p* = .047). While higher CZ amplitude differences were related to higher scores on BAS reward responsiveness (*r* = .518, *p* = .028), BAS-Drive (*r* = .718, *p* = .001), and greater amounts of left cortical activity (F4–F3 electrode pair, *r* = .540, *p* = .021). This seems to suggest that the P3 component in the low expectancy condition is related to more negative trait measures, while the midline electrode differences were related to individual variations in the positive trait measures reward responsiveness, appetitive drive, and left cortical activity. In the high win expectancy condition, amplitude differences for the FZ electrode were significantly related to higher amounts of left cortical activity (F4–F3 electrode pair, *r* = .547, *p* = .019).

As FN amplitudes are hypothesized to be related to measures of reward sensitivity, negative affectivity, and frontal asymmetry measures in favor of greater relative right frontal baseline activity, BAS reward responsiveness and STAI scores were selected and entered simultaneously into a multiple linear regression model for predicting FN amplitude differences for those electrode locations identified as statistically significant in initial hypothesis testing (low expectancy FZ and FCZ). In this case, none of the personality models were significant. This suggested that the FN was relatively independent of the measured personality variables; however, due to the overall low power of the analysis because of the small sample sizes needed for the primary ERP analyses, and the higher chance of a type II error, negative findings were not interpreted.

In addition, multiple linear regression models were computed for predicting individual differences in the P3 response amplitude differences between the high and low win expectancy conditions identified as significant in the earlier hypothesis testing (FZ, FCZ, and CZ electrodes). If the P3 differences were related to increased levels of affect responses to the expectancy violations in the high expectancy condition, as suggested by their correlations between personality measures and cortical asymmetry scores, P3 amplitudes were hypothesized as being significantly predictable by a weighted linear combination of personality scores.

Due to multicollinearity, F4–F3 was selected as an anterior asymmetry predictor, STAI-T as a personality predictor and baseline negative affect were selected and entered simultaneously into the regression model, as shown in Table 2. Of the predictor variables, only F4–F3 (*p* < .01) and STAI-T (*p* < .05) had significant zero-order correlations with FZ P3 amplitude difference scores between expectancy conditions, and both had significant partial effects in the full model. The three-predictor model was able to account for 60 % of the variance in P3 amplitude differences for the FZ electrode, *F*(3, 14) = 7.057, *p* = .004, *η* ^*2*^ = .60. For FCZ, a model composed of identical predictors was not as good a fit, but was still able to account for 46 % of the variance in P3 amplitude difference *F*(3, 14) = 4.01, *p* = .03, *η* ^*2*^ = .46. When applied to CZ, the model fit was again reduced, accounting for 27 % of the variance in P3 difference amplitude, although this time the overall model was not significant, *F*(3, 14) = 1.72, *p* = .209, *η* ^*2*^ = .27. Overall, this suggests that individual differences in P3 amplitudes between the high and low expectancy reward conditions may be predicted by personality variables related to cortical asymmetry and negative affect, but that the model shows a better fit with more anterior midline electrodes than central midline electrodes. Thus, an individual’s state negative affect and trait anxiety predicted more similar loss processing between expectancy conditions, such that unexpected and more expected loss–win feedback differences showed similar processing, while greater relative left cortical activity predicted greater differences in feedback processing by expectancy conditions (i.e., that unexpected loss feedback showed increased P3 amplitude than more expected losses).

**Table 2:** Individual differences in FZ P3 amplitude difference related to measures cortical asymmetry, affect, and personality (*N* = 18)

Variable	Zero-order *r*				*β*	*sr* ^*2*^	*B*
	STAI-T	F4–F3	NA-PRE	P3 DIFF			
NA-PRE				−.097	.17	.02	.260
F4–F3			−.263	.614**	.64**	.38	33.09
STAI-T		−.045	.208	−.473*	−.48*	.21	−.427
					Intercept = .198		
Mean	34.17	.163	13.5	−5.46			
SD	7.79	.133	4.53	6.92	*R* ^2^ = .602**		

* *p* < .05, ** *p* < .01

With the previous findings of the closer relationship of more central midline P3 amplitude with measures of appetitive motivation, a separate regression model for predicting CZ P3 differences was constructed and is shown in Table 3. F4–F3 was retained from the previous model and had a non-significant zero-order negative correlation with P3 CZ amplitude difference (*p* = .30), while baseline negative affect was replaced by baseline positive affect, which also had a non-significant negative zero-order correlation with P3 CZ amplitude difference (*p* = .355), and trait anxiety was replaced with BAS-Drive, which had a significant negative zero-order correlation with P3 CZ amplitude difference scores (*p* < .01). The three-predictor model was significant and was able to account for 42 % of the variance in P3 amplitude difference for the CZ electrode, *F*(3, 14) = 3.349, *p* = .049, *η2* = .42. This suggests that the P3 amplitude differences for the central midline electrode showed little difference between high and low expectancy conditions for individuals with higher positive affect, higher drive to receive reward, and increased left cortical activity.

**Table 3:** Individual differences in CZ P3 amplitude difference related to measures cortical asymmetry, affect, and personality (*N* = 18)

Variable	Zero-order *r*				*β*	*sr* ^2^	*B*
	BAS-D	F4–F3	PA-PRE	P3 DIFF			
PA-PRE				−.094	−.15	.02	−.22
F4–F3			.108	−.133	.07	.01	3.96
BAS-D		.279	−.081	−.627**	−.66**	.39	−2.1
					Intercept = 21.9		
Mean	11.5	.163	32.06	−8.64			
SD	2.431	.133	5.55	7.78	*R* ^2^ = .42*		

* *p* < .05, ** *p* < .01

## Discussion

The major findings from this study support our initial null hypothesis regarding FN amplitude differences; there were no significant differences in amplitudes with varying levels of win expectancy in a task without induction of positive affect responses by a boring task without semblance of gambling or monetary reward. Our findings support the conceptualization of the FN as a phasic suppression effect resulting from coding valence of outcomes as proposed in early studies of feedback processing [24, 28, 29]. The presentation of negative feedback in our fixed reward magnitude task induced an FN-type negative deflection occurring in the 225–325 ms post feedback time window. However, as hypothesized, the FN amplitudes did not significantly differ between high and low win expectancy. This deviation from the previous literature, and resultant hypothesis is based on the premise that previous studies have not necessarily accounted for reward expectations and affect response. Indeed, while previous studies have found FN differences are related to objective reward probability outcomes [10, 16, 30], and relationship to reward responsiveness measures [45, 59], the nature of these tasks suggests that differences in FN responses could have been due to other factors, such as subjective reward expectations not matching objective probabilities due to fallacious reasoning, affect responses occurring following unexpected wins or losses of actual money, or a higher reward salience of more immediate reinforcement value in the feedback. Moreover, our findings are similar to that reported by Van den Berg and colleagues [58] who found no association between FN and reward responsiveness in a gambling task.

Our task was not able to completely limit emotional responsivity, demonstrated through an overall decrease in subjective perception of affect through reduction in positive affect in the participants. Although this was found to be related to participant age and undergraduate class status, the reduction in affect was not intended by our task design. Although affect reduction limits our ability to interpret the results as affectively neutral, it does allow us to identify that violation of subjective reward expectations did not lead to increases in FN amplitudes in the absence of positive affective responses. This supports the previous findings of reduced FN amplitude in tasks involving no response choices and low subjective involvement in tasks [52], and implicates the possible role of affective information influencing overall ACC activation and FN responsiveness in tasks likely to induce affective responses. The overall reduction in positive affect might not suggest a lack of motivation on the part of the participants, as participant task-related accuracy was over 90 %, including the trials where correct response patterns were not yet known.

Utilizing the personality and cortical asymmetry information provides some insight into the relationships between the ERP responses and behavioral markers theorized to represent the functioning of underlying neurobiological systems. The basic relationships between these measures provided additional support to and replication of previous research, as measures of appetitive motivation, positive affect, and greater relative left cortical baseline activation tended to positively correlate. Similarly, measures of inhibition and negative affect tended to positively correlate and negatively correlate greater relative left cortical baseline activation. Although none of the personality or cortical asymmetry measures significantly related to FN amplitudes, these analyses were underpowered and suggest the need for additional research designed for individual differences analyses. As we found significant relationships between the P3 potential and these measures, these results hint that FN within the pattern response learning task was less strongly related to personality, cortical asymmetry, or outcome probability and supports its role in signaling the deviation from reward expectation, regardless of probability, to allow engagement of more adaptive response strategies.

The surprising finding of positive deflection occurring around 400 ms in the post feedback time window provides some insight into interpreting the feedback-related processing which occurred in the task. The ERP was significantly different from zero in loss feedback processing in both the high and low expectancy conditions, although amplitudes were significantly higher for more unexpected losses. This task design presented the high win expectancy loss feedback as a single negative stimulus following a string of highly expected win stimuli. Thus, this loss feedback could have represented a novel stimulus that was meaningful in the task as a signal of deviation from expectancy, suggesting that this component represents a P3b ERP component, as suggested by Donchin’s [70] schema updating model. A second possibility is that negative feedback, particularly in the high win expectancy condition, signals a need for stopping ongoing response patterns, and that this component represents a need to update response selection, which again would represent a P3b type component, but closer to Verleger and colleagues’ [71] conceptualization of the P3b.

However, the presence of the significant component during the periods of guessing new response patterns, where the negative feedback occurred equally with positive feedback, suggests that this component is not a classic novel P3b response and may indicate other cognitive processes. This would remain as a signal for needing to change the response output initially selected to one that matches the provided feedback pattern. A third possibility remains that the presence of this component during differences between the conditions may represent a trait response to the more unexpected negative feedback.

When directly correlated with measures of personality and affect, the low expectancy condition P3 amplitudes were related to higher trait anxiety for the FZ electrode, and low expectancy P3 amplitudes related to reward responsiveness and increased drive to obtain reward for the CZ electrode. With the functional divisions of the cingulate cortex into anterior affective processing and dorsal response selection processing, these relationships highlight that the P3 component may reflect the summation of varied processes. Specifically, that the anterior electrodes are more closely related to a general tendency to experience increased anxiety, while the more dorsal electrodes are more closely related to variables that may impact response selection, such as increased appetitive drive which would presumably spurn greater focus on immediate response selection in order to maximize rewards. However, for the high expectancy condition, only higher relative left cortical baseline activity was positively related to P3 amplitude for the FZ electrode, which seems counterintuitive in that anterior cingulate processing has been linked to negative affective processing and greater left cortical activation has been linked to measures of positive affect. This could suggest that those participants who have a general propensity for experiencing positive affect showed a heightened negative response to more unexpected loss feedback. However, without identifying the generator of this component, these links with cingulate functional division processes remain speculative.

Some additional support for the possible affective nature of the P3 component comes from the reported multiple regression models. The anterior midline electrodes showed a good overall fit with a model consisting of a weighted linear combination of measures of cortical asymmetry, trait anxiety, and baseline negative affect, while more dorsal midline electrodes showed a good overall fit with measures of cortical asymmetry, baseline positive affect, and higher appetitive drive. While this does not suggest that a P3 type cognitive process is not occurring, it does suggest some individual differences in that process. If the P3 truly represents a context monitoring process, it appears that those individuals with higher anxiety and negative affect show reduced perception of differences in the subjective meaning of the losses within differing contexts. If the P3 represents a response selection and initiation process, this suggests that those individuals with a higher drive to receive reward feedback react equally strongly to loss-related feedback, as each loss would signal a need to modify planned response patterns in the current experimental paradigm, regardless of the expectancy of the feedback outcome.

Source localization techniques may aid in identifying presumed locations of component generation, but were not performed in the current study. While the FN has considerable support for ACC generation, the P3 waveform generated in the current study might have a different generator and reflect a different cortical process. While the current study was originally designed to stabilize within-task affect variation, affective measurements did not occur throughout the task. Use of pre-post affect measurement and periodic visual analog scales allowed for some information concerning overall affect variation; no affect responses were assessed on a trial-by-trial basis. Thus, participants may have experienced within task affect responsivity to consistent wins and unexpected losses, which stabilized prior to the visual analog assessment. Similarly, the current paradigm revealed some overall reduction in positive affect that although supports the lack of positive affect induced by the task, also suggests that our results include unexpected error from the affect variation.

Future research may seek to utilize variations in task design seeking to increase motivational salience without the inclusion of gambling and direct monetary reward, in order to further stabilize overall affect variation and allow more specific findings regarding FN variation due to task-induced affect responses. Future studies could attempt to directly compare affective and non-affective inducing tasks with a single participant group, looking for such FN variability. Future studies may also attempt to identify if inducing a P3b type response through a more classic oddball paradigm shows similar relationships with affective measures and cortical asymmetry. Finally, future research attempting to stabilize affect responsivity could include trial-by-trial affect assessment to verify that participant affect is not varying significantly within trial blocks.

Overall, the current study could not directly relate FN and P3 amplitudes to most measures of affective and cortical asymmetry, and suggests that these components predominantly represent basic performance monitoring cognitive processes. Information regarding the differences between high and low expectancy suggest that affective states and traits and baseline asymmetry may predispose differences in cognitive processing of loss-related feedback. The P3 showed sensitivity to participant win expectancy, and showed some relationships to trait anxiety and appetitive motivation measures, but these were more pronounced for low win expectancy. Individual differences in error-related processing were revealed in the differences in P3 processing between the two conditions, such that trait anxiety, state negative affect, and baseline cortical asymmetry predicted differences in the cognitive processing of loss-related feedback. This suggests that while the overall amplitudes of the components reflect cognitive processes, affective states seem to predispose some participants to process loss-related feedback equivalently, regardless of whether they perceived the losses as occurring while guessing or occurring after the establishment of a learned pattern of response.
